# *In vivo* evaluation of osseointegration ability of sintered bionic trabecular porous titanium alloy as artificial hip prosthesis

**DOI:** 10.3389/fbioe.2022.928216

**Published:** 2022-09-14

**Authors:** Xiaowei Bai, Ji Li, Zhidong Zhao, Qi Wang, Ningyu Lv, Yuxing Wang, Huayi Gao, Zheng Guo, Zhongli Li

**Affiliations:** ^1^ Medical School of Chinese PLA, Beijing, China; ^2^ Department of Orthopaedics, The First Medical Center, Chinese PLA General Hospital, Beijing, China; ^3^ Department of Orthopaedics, The 987th Hospital of Logistics Support Force of Chinese PLA, Baoji, China

**Keywords:** artificial joint, aseptic loosening, porous Ti6Al4V, bionic trabecular structure, osseointergration, stress shielding

## Abstract

Hydroxyapatite (HA) coatings have been widely used for improving the bone-implant interface (BII) bonding of the artificial joint prostheses. However, the incidence of prosthetic revisions due to aseptic loosening remains high. Porous materials, including three-dimensional (3D) printing, can reduce the elastic modulus and improve osseointegration at the BII. In our previous study, we identified a porous material with a sintered bionic trabecular structure with *in vitro* and *in vivo* bio-safety as well as *in vivo* mechanical safety. This study aimed to compare the difference in osseointegration ability of the different porous materials and HA-coated titanium alloy in the BII. We fabricated sintered bionic trabecular porous titanium acetabular cups, 3D-printed porous titanium acetabular cups, and HA-coated titanium alloy acetabular cups for producing a hip prosthesis suitable for beagle dogs. Subsequently, the imaging and histomorphological analysis of the three materials under mechanical loading in animals was performed (at months 1, 3, and 6). The results suggested that both sintered bionic porous titanium alloy and 3D-printed titanium alloy exhibited superior performances in promoting osseointegration at the BII than the HA-coated titanium alloy. In particular, the sintered bionic porous titanium alloy exhibited a favorable bone ingrowth performance at an early stage (month 1). A comparison of the two porous titanium alloys suggested that the sintered bionic porous titanium alloys exhibit superior bone in growth properties and osseointegration ability. Overall, our findings provide an experimental basis for the clinical application of sintered bionic trabecular porous titanium alloys.

## 1 Introduction

Arthroplasty is considered the gold standard surgical treatment for joint function recovery after trauma or severe osteoarthritis. Population aging (Badley and Crotty, 1995) and obesity (Crowson et al., 2013) have been identified as the major contributing factors to the increased prevalence of degenerative osteoarthritis, which subsequently significantly increased the demand for total hip arthroplasty (THA) and total knee arthroplasty (Singh et al., 2019). However, surgical failure may still occur in a few cases, leading to serious consequences and requiring revision surgery. Up to 25% of patients after arthroplasty require revision surgery, and approximately 7% of revision surgeries are performed within the first 8 years after arthroplasty (Ulrich et al., 2008). The 15-years survival rate of revision surgery is merely 69% (Ulrich et al., 2008). The mean total expense for the revision of total knee arthroplasty was $75,028.07 in the United States (Delanois et al., 2017). Aseptic loosening is among the most common causes leading to prosthesis failure, observed in approximately 18% of cases (Bozic et al., 2010; Kremers et al., 2012). Moreover, studies have suggested that the mismatch between the metal implant in the body and the elastic modulus of the surrounding bone tissues is one of the primary reasons for aseptic loosening, which subsequently causes stress shielding, and eventually leads to prosthesis failure. Thus, the research on artificial joint materials has primarily focused on two issues: 1) increasing the osseointegration of BII, and 2) reducing the difference in elastic modulus of BII, thereby reducing stress shielding.

Titanium and its alloys are the preferred biomaterials for implants. Compared with other biomaterials, such as stainless steel and cobalt-based alloys, titanium-based alloys have excellent biocompatibility, corrosion resistance, and a high strength-to-weight ratio (Liu et al., 2004; Niinomi et al., 2012). Although other biomaterials, such as ceramics and polymers, are used in implantable medical devices, they are either too brittle or do not have sufficient mechanical properties for desired purposes (Saini et al., 2015). The application of bioceramic coatings addresses both the problem of ceramic brittleness and increases the performance of implant osseointegration properties (Montazerian et al., 2022). Although studies on tantalum are gradually increasing, higher cost of tantalum and difficulty in manufacturing have rendered it underappreciated in clinical application (Balla et al., 2010; Helsen and Missirlis, 2010). In 1977, Branemark proposed the term ‘osseointegration’ to describe the ability of implants to form mechanical and functional interconnections with bone tissues (Brånemark et al., 1977). Unfavorable osseointegration and the lack of a strong and durable connection between the bone and the implant surface are the main contributors to aseptic loosening and are a possible primary reason creating the conditions for bacterial growth and infection (Hu et al., 2019). Similar to the concept of “race for the surface” proposed by Gristina, if host cells can reach and occupy the implant surface first, stronger osseointegration can be achieved, and a barrier against microbial adhesion and reproduction can be established (Gristina, 1987).

To maintain bone mineral homeostasis, bones require to constantly self-adapt and remodel. When the titanium implant adjacent to the bone has a higher stiffness, the bone undergoes stress shielding, which results from the reduction in bone physiological load and eventually leads to bone resorption and final implant loosening (Van Lenthe et al., 1997; Ridzwan et al., 2007). Reducing the difference in the elastic modulus of BII can reduce stress shielding. The elastic modulus of titanium alloy is 113 GPa (Brizuela et al., 2019), whereas those of human cortical bone and trabecular bone are approximately 30 and 1.5 GPa, respectively (Keaveny and Hayes, 1993; Zysset et al., 1999). It has been proposed that porous titanium alloy can improve osseointegration and reduce the elastic modulus of titanium alloy. Studies have demonstrated that the porous structure has significant potential in improving cell adhesion and osseointegration (Heinl et al., 2008; Teixeira et al., 2012; Markhoff et al., 2015; Dallago et al., 2018). However, the structure is porous, which decreases mechanical properties, such as elastic modulus, thereby reducing stress shielding in bones (Soro et al., 2018; Yan et al., 2019; Yu et al., 2020).

Thus far, for preparing porous materials, 34 methods have been reported (Naebe and Shirvanimoghaddam, 2016; Singh et al., 2017; Trueba, 2017), including selective laser melting (SLM) and powder metallurgy (PM) technologies. SLM is a high-yield 3D-printing technology. Through the layer-by-layer preparation method, high-precision irregular and complex structures, which have huge application potential in biomedicine, can be prepared (Pattanayak et al., 2011; Shirazi et al., 2015). PM (sintered) is a considerably useful and relatively simple technique for preparing porous implants. Compared with other existing methods, it is inexpensive and can reduce material loss (Nouri et al., 2010). The application of PM and space-holder materials provides a suitable method for obtaining a porous titanium structure (Torres et al., 2014). The materials used as space-holders include sucrose, sodium fluoride, sodium chloride, and polymer particles (Arifvianto and Zhou, 2014). Magnesium is also used as a porogen for preparing dental and orthopedic porous implants (Kim et al., 2013). However, the remnants of the space-holder materials may form impurities in the foam (Kim et al., 2013; Lee et al., 2014). To increase the bone ingrowth of metal implants, hydroxyapatite (HA) coatings are used on the titanium alloy surfaces. The HA coating is currently a commonly used osseointegration material in clinical practice, as it increases and activates bone ingrowth (Svehla et al., 2002; Gandhi et al., 2009; Chen and Thouas, 2015).

In this study, PM technology of the sintering process (Steven et al., 2015) and SLM (3D printing) were used to prepare fully porous titanium alloys. The clinical commonly used HA-coated titanium alloys were compared, and osseointegration under early mechanical load was implanted in beagle dogs. This study aimed to identify inexpensive sintered porous materials with superior bone-implant osseointegration ability, which were compared with the HA coating and 3D-printing technologies commonly used in clinical practice. A superior option for the development of artificial joints was determined.

## 2 Material and methods

### 2.1 Material preparation

**FIGURE 1:**
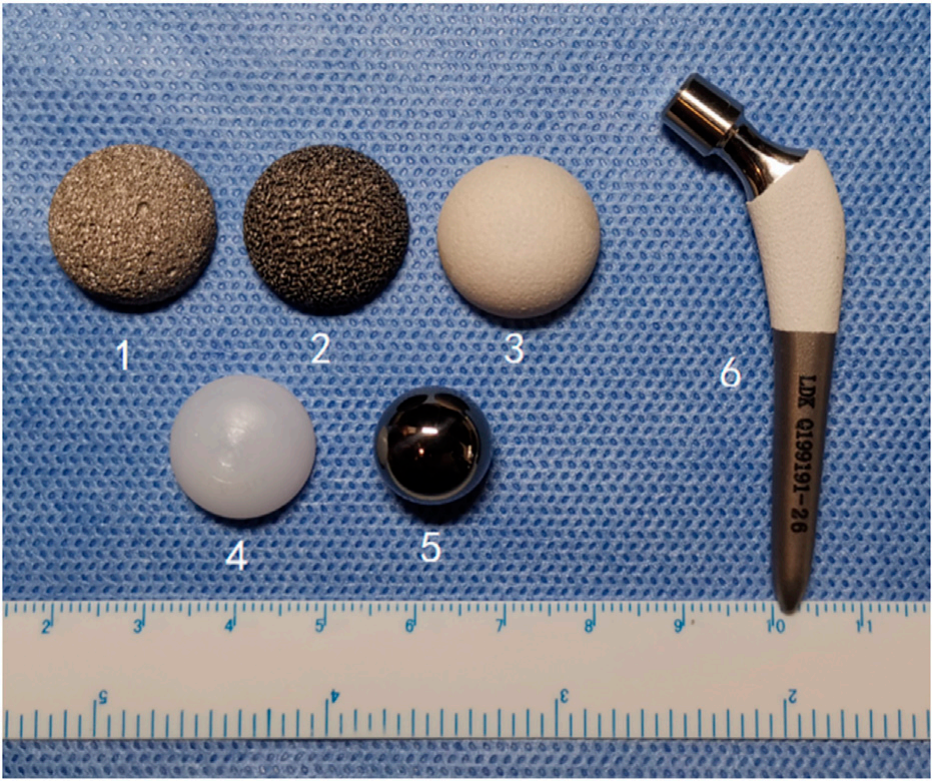
A complete set of hip prostheses in the experiment. (1, sintered porous titanium alloy acetabular cup; 2, 3D-printed porous titanium alloy acetabular cup; 3, HA-coated titanium alloy acetabular cup; 4. UHMWPE lining; 5. CoCrMo femoral head; 6. HA-coated titanium alloy femoral stem).

To conduct an experimental study, the hip prosthesis data of the beagle dogs were obtained *via* computed tomography (CT) scans from previous studies (Li et al., 2019a). According to the available prosthesis data, two porous titanium alloy acetabular cups (sintered and 3D printed) and HA-coated titanium alloy acetabular cup that is commonly used in clinical applications were prepared (Figure 1).

Preparation of sintered bionic bone trabeculae of fully porous titanium alloy (referred to later as sintered porous titanium). According to a newly discovered sintering process (Li et al., 2016), sintered porous titanium alloy materials made of titanium alloy powder were used. Titanium alloy powder (fineness ≥99.5%, and grain size <100 μm) was used as the initial material. In particular, Ti6Al4V powder, water, agar, ammonium alginate, and medical gelatin were mixed at 70°C for 6 min to obtain a fluid foam, which was then cast into a mold and cooled until it gelled. After demolding, the sample was dried at atmospheric pressure and room temperature, and then calcined to 500°C (20°C/h) at 10^–3^ Mbar pressure, maintained warm for 2 h, and then sintered at 10^–4^ Mbar pressure to 1,200°C–1,500°C (5°C/min) and maintained warm for 2 h more. The fully porous material with about 75% porosity was then prepared: a hemispherical hollow acetabular cup of 2 mm thickness; the material was divided into three types according to the diameter (18, 20, and 22 mm). The inner surface of the porous cup was smoothed using the smear technique. All the porous samples were sonicated to remove impurities. Thereafter, the porous samples were dried in a sterile environment. HA-coated titanium acetabular cups are prepared using the physical deposition technique of low temperature plasma spraying. According to the above-mentioned prosthesis design, the HA-coated acetabular cups with three diameters (18, 20, and 22 mm) and a thickness of 2 mm were also prepared. The thickness of HA coating was approximately 150 ± 50 μm.

The 3D-printed porous titanium alloy samples were fabricated using the SLM 250 HL device. The computer-aided design (CAD) technology was used to construct the required sample geometry. A cyclic process was used to build the scaffold layer by layer on a titanium alloy substrate platform. The cyclic process included applying a titanium alloy powder layer with an average particle size of 15–45 μm on the platform and laser irradiation to the selected point powder layer. The CAD-derived geometry led to the melting of the powder and its fusion with the underlying support layer. Finally, the platform was reduced by a one-layer thickness to enable the application of the next powder layer, and the cycle was repeated. A laser power of 200 W, a focal point with a laser spot diameter of 100 μm, scanning speed of 1,300 mm/s, and volume of 80 μm were used for the incubation distance. The system was operated in an overpressure argon environment, and the oxygen level in the processing chamber was <0.2%. Ultrasonic cleaning, drying, and heat treatment (1,400°C, 3 h) of all individual parts were conducted. Based on the findings of the previous work, a porous acetabular cup was prepared with a porosity similar to that of the cup obtained from the sintering process.

The SLM technology and spraying process were used to prepare the HA-coated artificial dog femoral stems of different sizes to match the femoral anatomy. Then, CoCr alloy femoral heads and ultra-high molecular weight polyethylene (UHMWPE) linings of different sizes were prepared to connect with the acetabular cup and femoral stem. Figure 1 shows a complete set of dog hip prosthesis components.

The sintered porous titanium alloy used in this study was provided by Zhongao Huicheng Company, and all the remaining prostheses and special surgical instruments were obtained from Beijing LDK Technology Co., Ltd. All the samples in the present study were sterilized in ethylene oxide before use. Finally, the disinfected samples were stored for 14 days to dissipate ethylene oxide.

### 2.2 Material characterization

An optical microscope (Olympus DP74, Olympus America Inc., United States) was used to observe the structure and surface morphology of the sample. A scanning electron microscope (FEI, XL30-FEG, United States) was used to qualitatively observe the micromorphology of the porous titanium alloy (surface morphology and connectivity). A suitable photo was captured to observe each sample from three selected fields of view; ten values were measured in each field, and the mean pore size was calculated. The calculation method of the porosity of each porous sample was as follows: the volume density (q) of the sample was determined by measuring the physical size and mass of the sample; the Archimedes principle was used to measure apparent density (q') in water. The metal volume fraction (VF) was calculated as follows: VF = q/q'. Porosity PP = 1−VF = 1−(q/q'). According to the method reported by Hotaling (Hotaling et al., 2015), the ImageJ software (National Institute of Health, Bethesda, United States) was used to calculate the wire diameters of the two porous materials.

### 2.3 Canine total hip arthroplasty

#### 2.3.1 Experimental design

The experimental design included three observation time points, i.e., one, three, and 6 months after the procedure. Three acetabular cups of different structural materials were compared. Experimental group one (*n* = 9) used sintered porous titanium alloy acetabular cup; experimental group two (*n* = 9) used 3D-printed porous titanium alloy acetabular cup, and the control group (*n* = 9) used clinically commonly used HA-coated titanium alloy acetabular cup. Only special professional instruments were used for THA. During the procedure, uniform polyethylene liners, CoCrMo ball heads, and HA-coated titanium alloy femoral stem prostheses were used (Figure 1).

The experimental process included: 1) animal THA and prosthesis implantation were conducted based on randomization; 2) observations were performed at 1, 3, and 6 months for sampling, and fluorescent labeling was used before sampling; 3) euthanasia, subsequent MicroCT analysis, fluorescence analysis, and histomorphological analysis were performed. According to a computer-generated randomized list (IBM SPSS Statistics®), nine beagles per material group were randomly assigned to three time points for observation, and a total of twenty-seven beagles were selected.

#### 2.3.2 *In vivo* animal experiments

Twenty-seven healthy adult beagle dogs (weighing 11–15 kg; 14 females and 13 males) provided by the Experimental Animal Center of Chinese PLA General Hospital were used for this experimental study. All the animals were housed in an environment with a temperature of 18°C–29°C, relative humidity of 30%–70%, and a light/dark cycle of 12/12 h and fed with standard food and water. All animal studies (including the euthanasia procedure) were performed in compliance with the ARRIVE guidelines. The study protocol was reviewed and approved by the Animal Ethics Committee of Chinese PLA General Hospital (ethics number: 2019-D15-17). Animal experiments were performed in the Animal Center of Chinese PLA General Hospital.

#### 2.3.3 Surgical procedure

**FIGURE 2:**
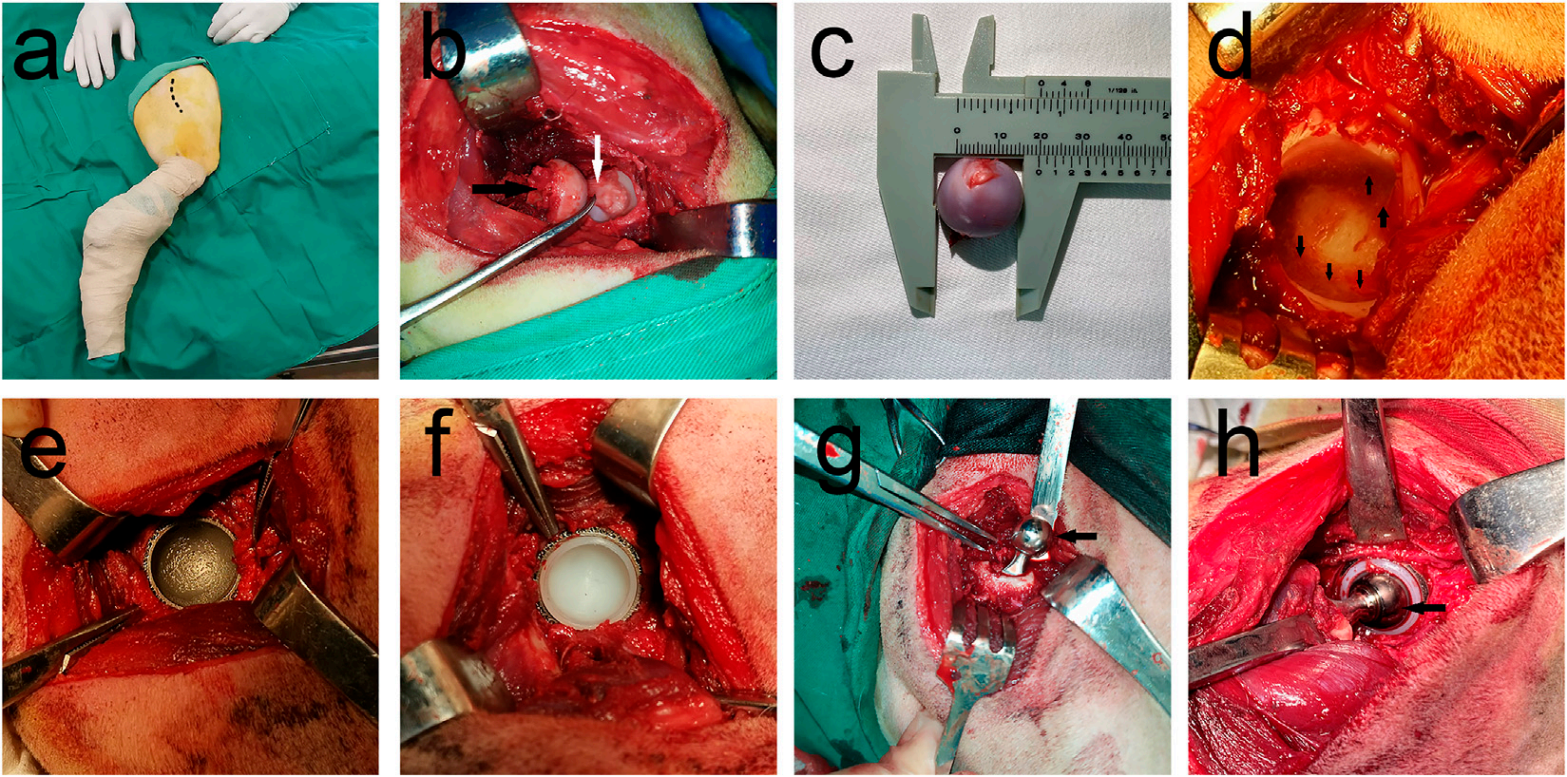
THA surgery procedure. **(A)** Preparation of surgical area, Dotted line shows the planned incision for surgery; **(B)** Femoral head removal using swing saw, Black arrow points to the resected femoral head and white arrow points to the unresected ligament of the head of femur; **(C)** Femoral head diameter measurement shows that femoral head diameter is approximately 20 mm; **(D)** Black arrow shows the articular surface after removal of articular cartilage, with good bleeding; **(E)** Installation of the acetabular cup, 3D-printed porous titanium mortar cup in a good position; **(F)** Complete installation of UHMWPE liner on metal acetabular cup, **(G)** Installation of the femoral stem and femoral head. Black arrow points to metal femoral head; **(H)** Joint repositioned and checked for motion. Installed femoral stalk is reinstalled into the acetabular fossa, and the joint movement was checked for obvious loosening and prosthesis dislodgement, favorable prosthesis movement tension, with black arrow denoting the metal femoral head.

First, 27 samples (9 of each material) were implanted in 27 beagle dogs. Unilateral THA was performed on all beagles. The previous experimental surgical procedure (Li et al., 2019a) was referred and the forelimb was first anesthetized *via* intravenous injection of 2% pentobarbital sodium solution (30 mg/kg). Surgery was performed using a lateral approach (Figure 2A) to bluntly separate the subcutaneous tissues and articular capsule to expose them to the level of the lesser trochanter. Then, a longitudinal incision of the articular capsule was conducted to protrude the femoral head. A pendulum saw was used to perform osteotomy along the longitudinal axis of the femur, and then, osteotomy was continued from the lower edge of the bone crest incision to the lesser trochanter (Figure 2B). Measurement of the femoral head diameter and selection of a suitable prosthesis (Figure 2C). The acetabulum was exposed, and suitable acetabular file size was used to remove the soft tissues and acetabular cartilage until there was uniform exudation (Figure 2D). The metal acetabular cup was placed into the acetabulum after grinding (Figure 2E). Subsequent fitting of a suitable UHMWPE liner to the metal acetabular cup (Figure 2F). After opening and expanding medullas, the femoral stem was implanted, and the femoral head prosthesis was installed (Figure 2G). The traction of the femur was performed in the abduction position; the femoral head was reduced; the hind limbs were moved to determine whether the range of motion was ideal (Figure 2H). After ensuring that no issues remained to be addressed, the muscle and skin were sutured layer by layer.

After the procedure, the dogs were separately fed and intramuscularly injected at a dose of 3 g/day of penicillin for 7 days. X-ray observations were immediately performed after the procedure to assess the position of the prostheses in all the dogs, and subsequently at 1, 3, and 6 months after the procedure to assess the status of the prostheses.

#### 2.3.4 Fluorescent labeling

The animals were injected with fluorescently labeled substances before sampling, and the beagle dogs were administered intramuscular injections of tetracycline (10 mg/ml, Sigma, United States) at a dose of 30 mg/kg for 2 weeks before the animals were euthanized. 4 days before the dogs were euthanized, they were administered an intramuscular injection of calcein (10 mg/ml, Sigma, United States) at a dose of 10 mg/kg. After sampling, the distance between the internal bone of the prosthesis and double immunofluorescent labeling of the bone around the prosthesis was measured at least five times, and the average length of this distance was calculated and divided according to the 10-days labeling interval, which was the bone mineral apposition rate (MAR, μm/d).

#### 2.3.5 MicroCT analysis

At 1, 3, and 6 months after the implantation, 27 beagle dogs (9 beagle dogs at each time point) were euthanized by injecting an overdose of anesthetic solution (pentobarbital sodium). All the specimens were soaked in a 4% formaldehyde solution after sampling. Before pathological sectioning, high-resolution micro-CT scanning (Quantum GX2, PerkinElmer, United States) was performed on all the specimens. The X-ray source was set to 70 Kv and 114 μA. The scanning was performed on a 360° rotation, and the images were acquired every 1°. The Data Viewer software (Quantum GX2, PerkinElmer, United States) was used to evaluate the reconstructed image, and it was rotated to ensure the perfect alignment of the implant. A volume of interest (VOI) with a thickness of 3 mm was selected for all the samples, including the 2 mm metal acetabular cup and 1 mm bone tissue in the peripheral cup. After selecting VOI, the CTAn software (Quantum GX2, PerkinElmer, United States) was used to analyze the data. First, a 3 mm-thick hollow hemispherical VOI was selected. Then, local adaptive thresholding was used to segment the image and select the optimal threshold parameters for the bones and implants. The following results were measured (Bruker, 2015): implant volume and bone volume (%), and it was expressed as bone volume/tissue volume (BV/TV, %).

#### 2.3.6 Histomorphological analysis

After the specimens were fixed in 4% formaldehyde for 7 days, they were dehydrated in the order of ethanol concentration (50%, 60%, 70%, 80%, 90%, and 100%); the samples were incubated in each concentration for 72 h. They were then embedded in a methyl methacrylate solution without decalcification and were polymerized at 37°C for 1 week. Then, the samples were cut using a modified interlocking diamond saw (EXAKT, Norderstedt, Germany) and ground into 20 μm sections. Next, a confocal laser microscope (Leica, Germany) was used to observe the fluorescent dye labels. The bone mineral apposition rate (MAR, the vertical separation between two fluorescent dye labels/injection intervals) was analyzed from the fluorescent dye-labeled image, which usually indicates the growth rate of new bone. After fluorescence analysis, the sample was stained with methylene blue (MB)/acid fuchsin (AF). Microscopic analysis was performed using an optical microscope (Olympus DP74, Olympus America Inc., United States) connected to a digital camera. The percentage of direct contact between the new bone and implant surface (bone-implant contact, %BIC) was determined using the OM-BDHS 3.3.0.2 software (OM-HRDVS, OSTEOMETRICS, INC., United States). The average BIC of all implants in each group was calculated and statistically compared.

#### 2.3.7 Statistical analyses

Statistical analyses were performed using the Statistical Package for Social Sciences software (SPSS 22.0, United States); the graph was plotted using the SPSS or GraphPad software (United States), and the results were presented as mean ± standard deviation (S.D). Statistical differences were assessed using the *t*-test or one-way analysis of variance. *p <* 0.05 was considered statistically significant.

## 3 Results

### 3.1 Material characteristics

**FIGURE 3:**
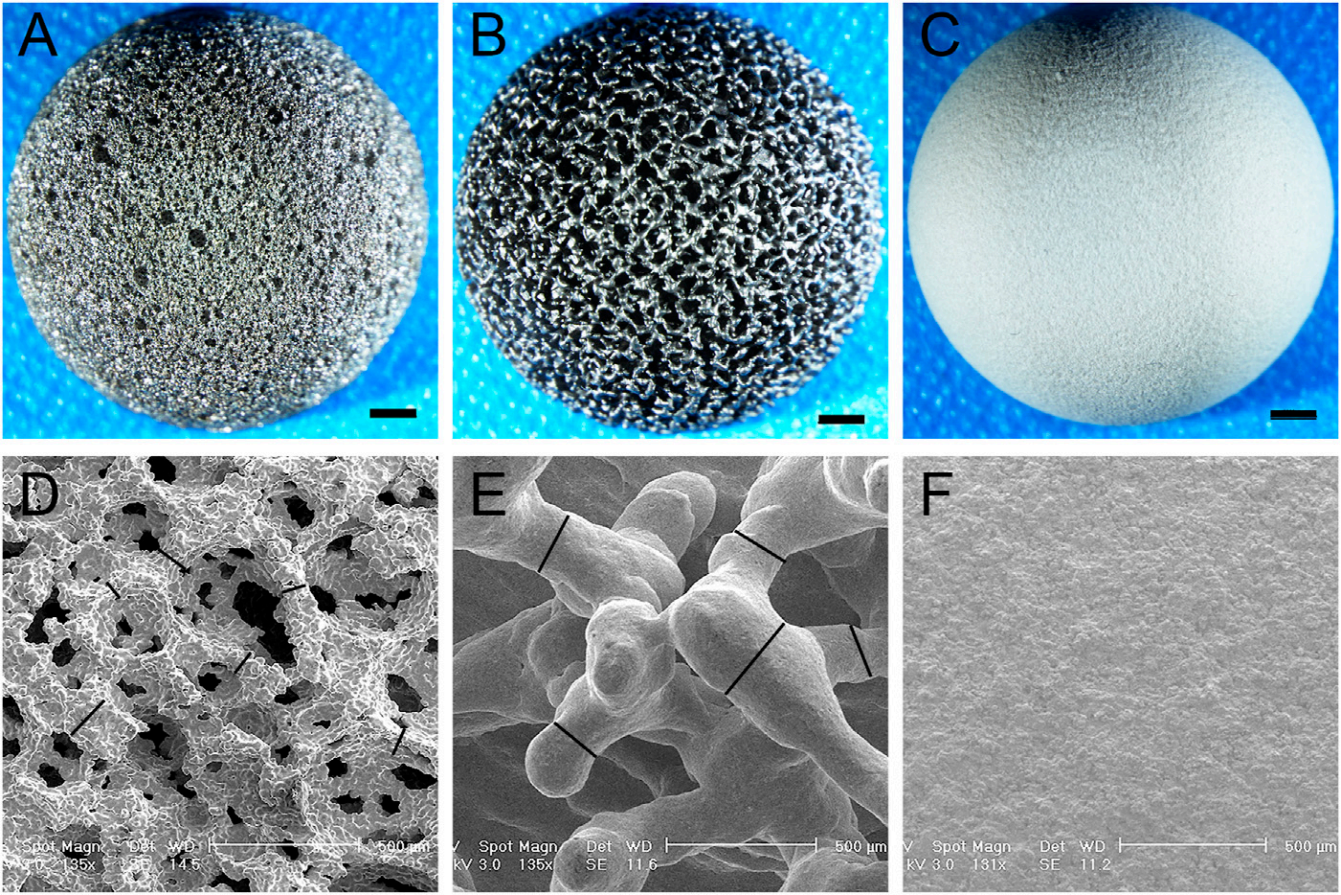
Light and electron microscopy images of the acetabular cup of three materials. **(A–C)** Light microscope images of the acetabular cup of three materials at 2 mm scale. **(A)** Sintered porous titanium alloy acetabular cup; **(B)** 3D-printed porous titanium alloy acetabular cup, and **(C)** HA-coated titanium alloy acetabular cup. **(D–F)** Electron microscopy images of the acetabular cup of three materials, the scale is 500 μm. **(D)** Sintered porous titanium alloy acetabular cup. A spatial porous structure similar to cancellous trabeculae can be observed, the black lines are marked as wire diameter in the figure; **(E)** 3D-printed porous titanium alloy acetabular cup. The black lines are marked as wire diameter, comparison with a wire diameter of sintered porous titanium alloy *p* < 0.001. **(F)** HA-coated titanium alloy acetabular cup. See that surface is covered with dense HA particles.

The sintered and 3D-printed fully porous titanium alloy acetabular cups were used, as shown in Figure 3. Their material properties are shown in Table 1. The observation conducted under a light microscope and electron microscope revealed that the porosities of the 3D group and the sintered group were 75.2% ± 1.40% and 74.3% ± 3.45% (*p >* 0.05), respectively, and the pore diameter of the two groups were 532.2 ± 71.43 µm and 515.3 ± 199.49 µm (*p >* 0.05), respectively. The wire diameters of the porous materials of the 3D and sintered groups were 224.15 ± 32.38 µm and 60.90 ± 22.87 µm (*p <* 0.001), respectively.

**TABLE 1:** The numerical differences of the physical parameters of the porous structures in the two groups.

	3D	Sintered
Porosity	75.2 ± 1.40%	74.3 ± 3.45%
Pore size	532.2 ± 71.43 µm	515.3 ± 199.49 µm
Wire Diameter*	224.15 ± 32.38 µm	60.90 ± 22.87 µm
Elastic modulus*	4.46 ± 0.38 GPa	1.77 ± 0.28 GPa
Compressive strength*	186.73 ± 16.03 MPa	97.10 ± 7.52 MPa

*Represents statistically significant differences in data between the two groups (*p <* 0.001).

For the physical properties of the two materials in the 3D group and sintered group, according to previous studies (Li et al., 2019b), the elastic moduli of the porous titanium alloy were 4.46 ± 0.38 and 1.77 ± 0.28 GPa (*p* < 0.001), and the compressive strength was 186.73 ± 16.03 and 97.10 ± 7.52 MPa (*p <* 0.001), respectively. Therefore, after testing, the physical properties of the two materials were stable and homogeneous and met the basic requirements of animal experiments.

### 3.2 Canine total hip arthroplasty

All the experimental animals were uniformly managed after the procedure. There were no significant changes in the food and water intake and body weight among different groups. No death or marked clinical complications (such as infection or drug complications) were observed during the follow-up period.

#### 3.2.1 Radiology findings

**FIGURE 4:**
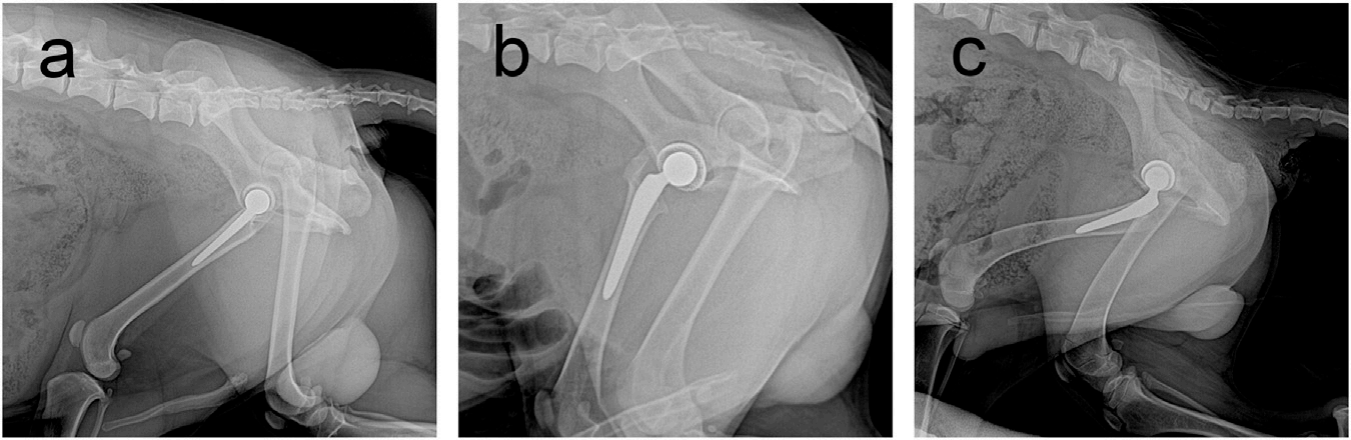
Follow-up X-rays of prostheses of three materials at 6 months postoperatively. Prosthesis can be seen in a good position, with no obvious loosening and dislocation and other performance. **(A)** Sintered porous titanium alloy acetabular cup groups; **(B)** 3D-printed porous titanium alloy group; **(C)** HA-coated titanium alloy acetabular cup group.

At the final follow-up, the X-ray film confirmed that the prosthesis was in a normal position, and no obvious dislocation or loosening was observed (Figure 4). Owing to numerous pores, the porous acetabular cup showed a slightly higher transmittance under X-rays than the HA-coated cup. No transparent zone was observed around the three groups of acetabular cup prostheses. A slight increase in bone density was observed at the top of the femur and acetabulum, and there was no significant difference in bone density around the three groups of prostheses (*p* > 0.05).

#### 3.2.2 Micro-CT

**FIGURE 5:**
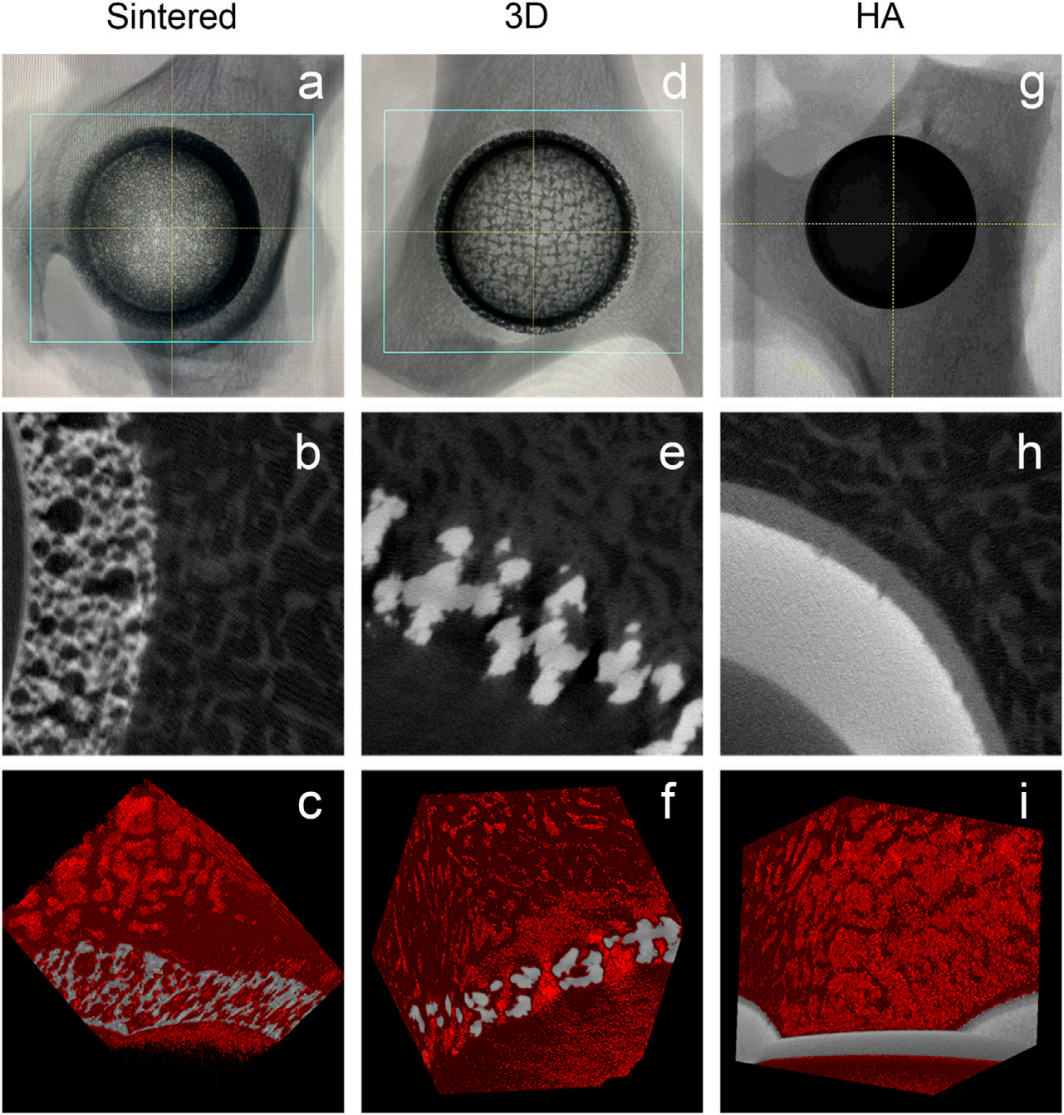
MicroCT images of different materials during the follow-up for 6 months. **(A–C)** Sintered porous titanium alloy acetabular cup; **(D–F)** 3D-printed porous titanium alloy acetabular cup; **(G–I)** HA-coated titanium alloy acetabular cup. a, d, and g are the direct specimen imaging of the MicroCT scan; b, e, and h are the two-dimensional specimen imaging of the MicroCT scan. In the figure, the gray-white is the titanium image, and the gray-black is the bone trabecular structure; c, f, and i are the specimen 3D reconstruction imaging of the MicroCT scan. Titanium alloy image is in gray, and the 3D reconstruction image of bone trabeculae is in red.

All specimens were fixed with formaldehyde and observed using Micro-CT scanning. Figures 5A,D,G show that the acetabular cup was correctly inserted into the acetabulum during the procedure. Figure 5 shows that there was a large amount of bone tissue surrounding the acetabular cup, and there no distinct bone loss around the implant is observed; furthermore, favorable integration between the bone tissue and implant is observed. As shown in Figures 5B,C,E,F, the bone tissues around the implant and ingrown bone tissues can be observed. These images show the osseointegration in the porous structure. In the control group, massive bone tissues are also observed around the HA-coated titanium alloy acetabular cup, but no new bone is observed to grow into the implant (Figures 5H,I).

**FIGURE 6:**
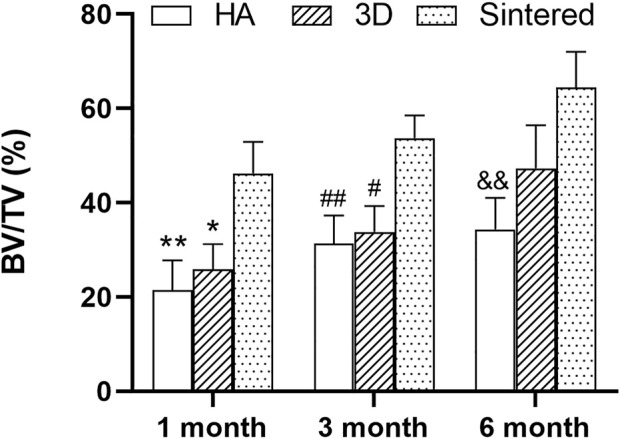
BV/TV analysis of three materials at different times. *p < 0.05 when comparing 3D group and sintered porous titanium alloy group at 1 month; **p < 0.01 when comparing HA group and sintered porous titanium alloy at 1 month; ^#^fn# *p* < 0.05 when comparing 3D group and sintered porous titanium alloy group at 3 months; ^##^fn##*p* < 0.01 when comparing HA group and sintered porous titanium alloy group at 3 months; ^&&^fnamp *p* < 0.01 when comparing HA group and sintered porous titanium alloy group at 6 months.

After comparing the BV/TV values of the three materials in different periods after MicroCT analysis (Figure 6), the sintered porous titanium alloy acetabular cup had a higher volume fraction ratio than that of the 3D-printed titanium alloy acetabular cup group and the HA group at 1 and 3 months. At 6 months, no difference between the 3D printed group and the sintered porous titanium alloy group was observed (*p* > 0.05), whereas *p <* 0.01 was observed between the sintered group and the HA group.

#### 3.2.3 Fluorescence staining analysis

**FIGURE 7:**
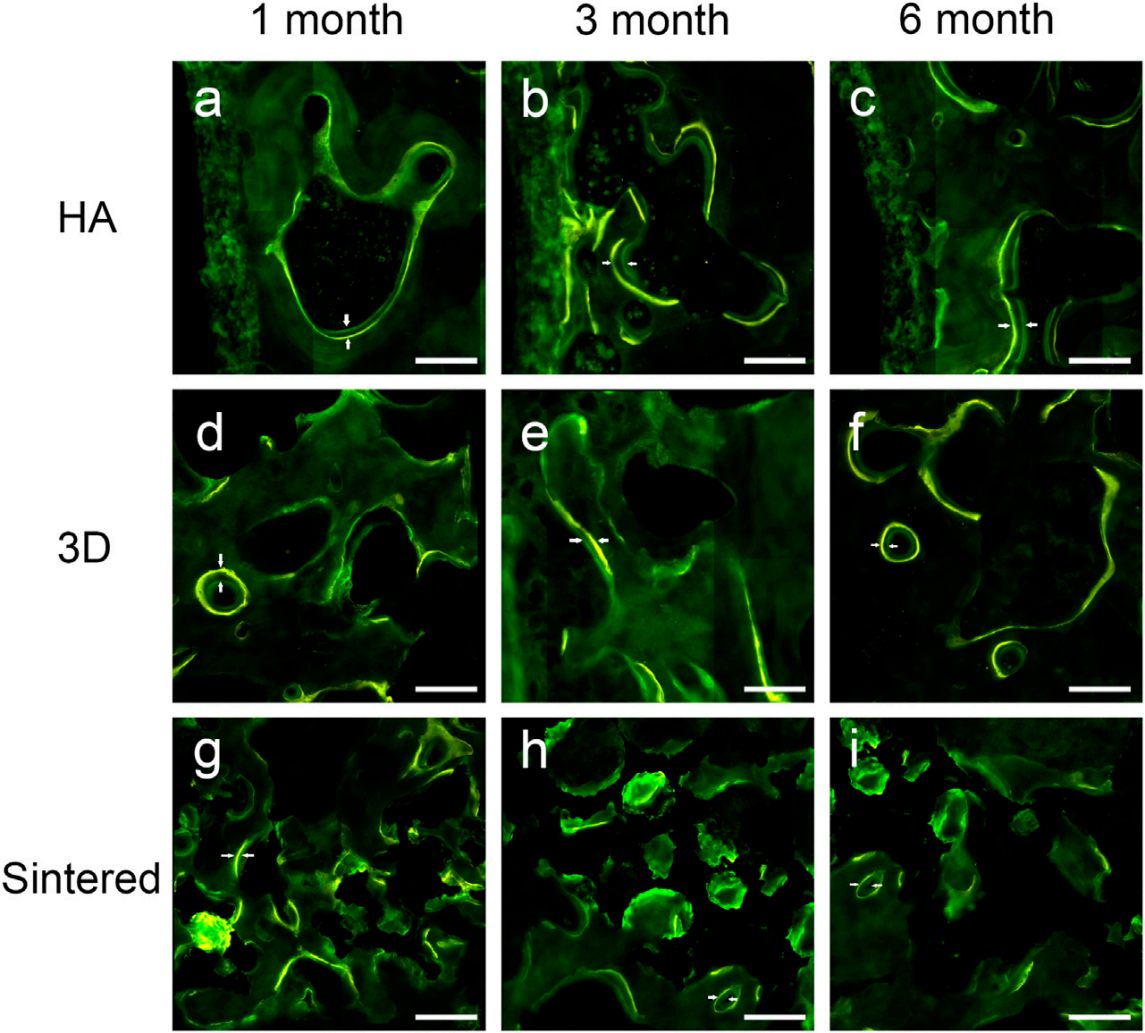
Fluorescently labeled photographs of the three materials at 1, 3, and 6 months of follow-up. (A–C) The fluorescently labeled photos of HAcoated titanium alloy acetabular cup at 1 (A), 3 (B), and 6 (B) months. (D–F) Fluorescently labeled photos of 3D-printed porous titanium alloy acetabular cup at 1 (d), 3 (E), and 6 (F) months. (G–I) Fluorescently labeled photos of sintered porous titanium alloy acetabular cup at 1 (G), 3 (H), and 6 (I) months. The scale was 200 μm. Double white arrows in the figure denote the fluorescence marker for new bone growth interval.

A confocal laser microscope (Leica, Germany) was used to observe the fluorescent dye labels. Verification of bone reconstruction was performed *via* intermittent fluorescent bone labeling and new bone deposition. The labels of new bone formation were detected in the pores of the two porous implants (Figure 7, tetracycline showed yellow, and calcein showed green).

**FIGURE 8:**
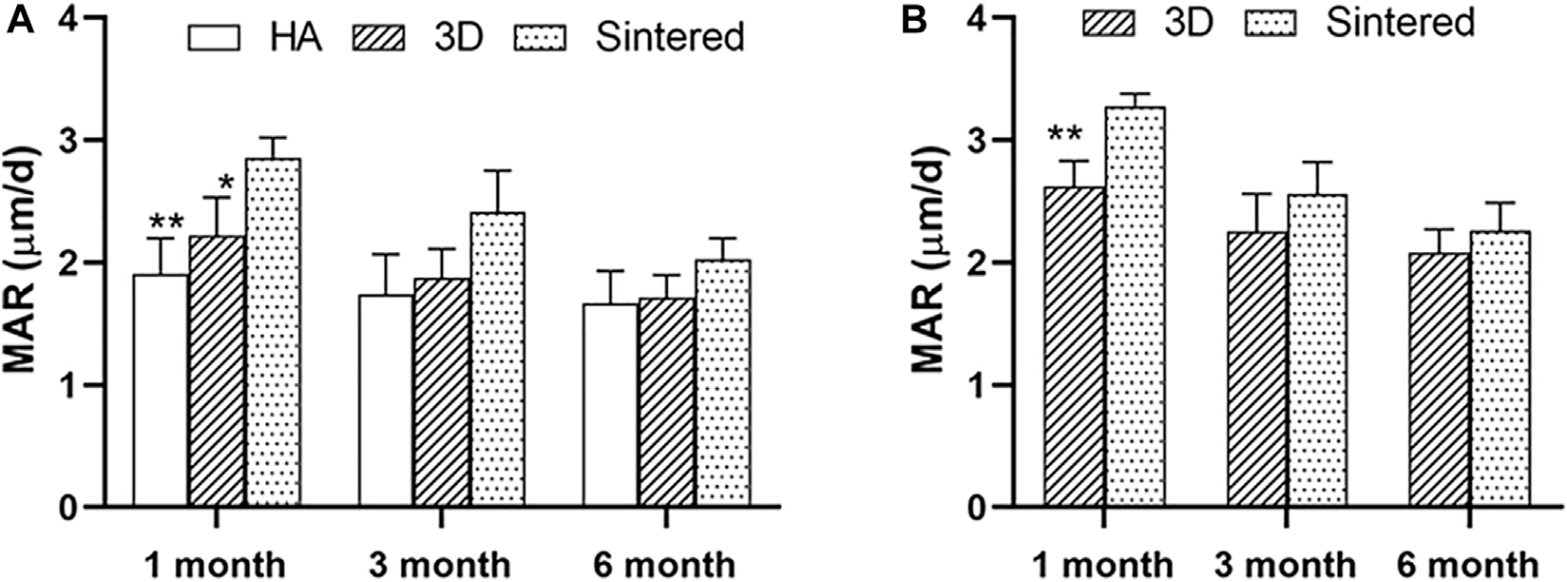
Comparison of MAR values around prosthesis and MAR values in prosthesis with different materials. **(A)** The MAR values around the prosthesis of three materials at different times. * indicates that MAR values of the sintered group and 3D group were significantly different at 1 month, p < 0.05; **, indicates that MAR values of sintered group and HA group were significantly different at 1 month, p < 0.01. **(B)** MAR values in prosthesis of two porous materials at different times. *, indicates that the MAR values of the sintered group and 3D group were significantly different at 1 month, p < 0.01.

At months 1, 3, and 6, greater MAR values were measured inside the porous structures of both porous materials than around the acetabular cups (Table 2). Figure 8A shows a comparison of the MAR values of the three groups. The sintered group had the highest MAR value at 1 month, which was higher than those of the HA-coated (*p* < 0.01) and 3D-printed groups (*p* < 0.05). The MAR of the two porous materials was higher than that of the HA-coated alloy at months 3 and 6; however, no statistical difference was observed among the groups; the MAR of each group gradually decreased with increasing time. Figure 8B shows the differences in the MAR value between the two porous groups within the prosthesis; again, at 1 month, the MAR value of the inward bone growth was greater in the sintered group than in the 3D group (*p* < 0.01); at months 3 and 6, the sintered group performed better than the 3D-printed group, however, there was no statistical difference between the groups. The MAR value of sintered porous titanium was higher than the other groups both within and around the prosthesis, indicating that the structure of sintered porous titanium promotes bone ingrowth, which was particularly evident in the first month.

**TABLE 2:** MAR values of different materials around and inside the acetabular cup prosthesis (μm).

	Around the prosthesis			In the prosthesis	
	Sintered	3D	HA	Sintered	3D
At 1 month	2.86 ± 0.16	2.22 ± 0.31	1.91 ± 0.29	3.28 ± 0.10	2.62 ± 0.21
At 3 months	2.41 ± 0.34	1.87 ± 0.24	1.74 ± 0.33	2.56 ± 0.26	2.25 ± 0.31
At 6 months	2.03 ± 0.17	1.71 ± 0.19	1.67 ± 0.26	2.26 ± 0.23	2.08 ± 0.19

#### 3.2.4 Histomorphological analysis

**FIGURE 9:**
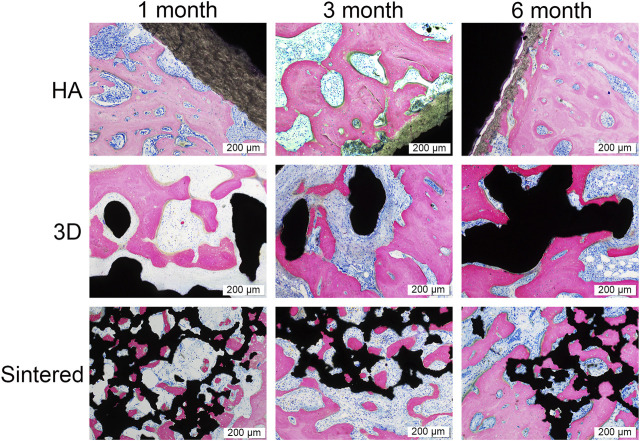
Pathological images of the three materials at 1, 3, and 6 months. Black in the picture represents the titanium alloy metal and the bone tissue is stained by rosy red. The scale was 200 μm. In the HA group, there was a progressive decrease in HA coating resorption and a progressive increase in peripheral bone tissue with time. At month 1, both the 3D and sintered groups showed more osseointegration than the HA group. The sintered group has more bone ingrowth into the porous structure in the sintered group than in the 3D group because of the more bionic 3D structure of the sintered group, and the bone tissue was better integrated with the sintered porous structure. As time increased, more bone tissue was observed surrounding the titanium cup in all three groups.

The experimental materials were well integrated with the surrounding tissues during the three experimental time points. No signs of inflammation, necrosis, or fibrous reaction were detected in the vicinity of the implant. The newly formed bone was in direct contact with the implant, filling the surface pores that were continuous with the natural bone around the recipient site. The new bone comprised mature bone trabeculae, arranged in layers in contact with the surface material (Figure 9). The trabecular bone tissue around the prosthesis had a regular pattern and morphology.

**FIGURE 10:**
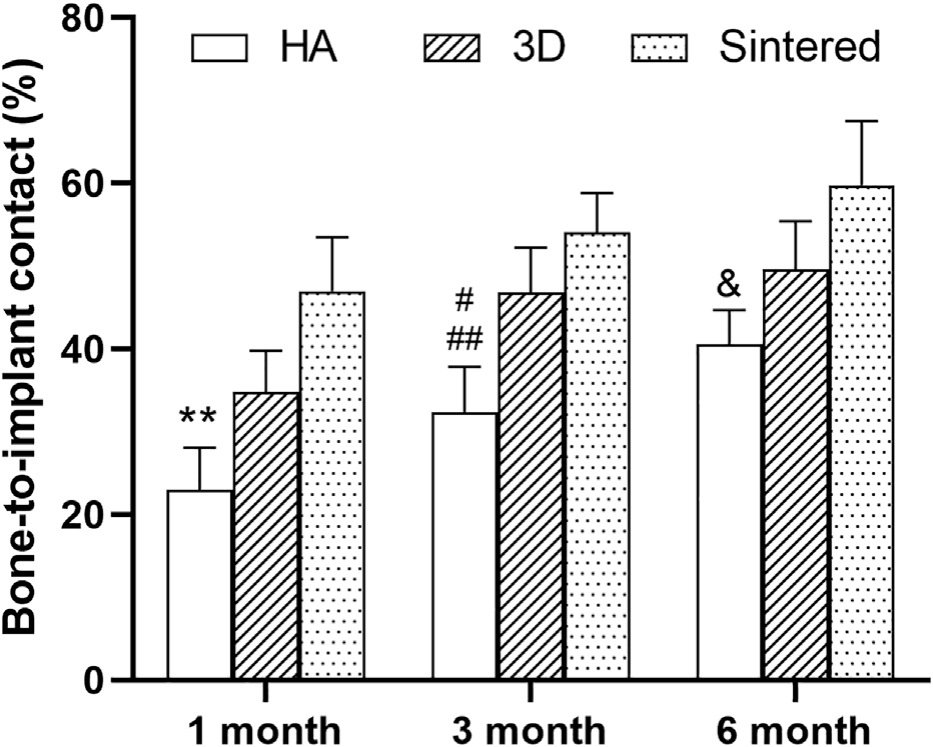
BIC values of three materials over time. **, comparison of HA and sintered porous titanium at 1 month, *p* < 0.01; ^#^, comparison of HA group and 3D porous titanium group at 3 months, *p* < 0.05; ^##^, comparison of HA group and sintered porous titanium group at 3 months, *p* < 0.01; &, comparison of HA group and sintered porous titanium group at 6 months, *p* < 0.05.

The hard tissue sections were stained with MB/AF to compare the bone ingrowth around the prosthesis of several materials and to analyze the %BIC. The %BIC of each material increased with time. At the same time point, the differences among different materials are shown in Figure 10. At 1, 3, and 6 months, the sintered porous titanium alloy exhibited a higher BIC value than the HA-coated titanium alloy acetabular cup. At 3 months, the 3D-printed group exhibited a higher BIC value than the HA group (*p <* 0.05), and the sintered group also exhibited a higher BIC value (*p <* 0.01) compared with the HA group. The BIC values of the sintered porous titanium alloy acetabular cup also gradually increased at three time points, but there were no significant differences among them (*p* > 0.05). At 6 months, the sintered porous titanium alloy acetabular cup exhibited a higher BIC value than the HA-coated acetabular cup (*p <* 0.05). The HA-coated titanium alloy acetabular cup exhibited a higher BIC value at 6 months than at 1 month (*p <* 0.05).

## 4 Discussion

The osseointegration of BII represents an important factor affecting the *in vivo* survival life of the artificial joint prosthesis. To encourage inward bone growth, the prosthetic surfaces have been treated with metallic titanium fibers, titanium plasma spraying, and sandblasting for improving the titanium surface roughness and porosity. These efforts were aimed at improving the stability of the initial component and consequently reducing the incidence of aseptic loosening of the artificial joint (Yoshioka et al., 2018). New technologies have facilitated the introduction of highly porous metals that enhance osseointegration and reduce stress shielding (Gallart et al., 2016; Hosny et al., 2018). Studies have demonstrated that the widespread use of highly porous titanium and alloy acetabular cups in primary hip replacements significantly reduces revision rates (Malahias et al., 2020). Moreover, the application of fully porous materials will further improve the osseointegration rate (Cartmell et al., 2003; Wang et al., 2005).

In this study, we used a bionic trabecular fully porous titanium alloy acetabular cup fabricated using a sintering process and compared its performance with 3D-printed porous titanium alloys and HA-coated titanium cups in terms of their osseointegration ability in total hip replacement of beagle dogs. The results demonstrated that both sintered bionic porous titanium and 3D-printed titanium alloys exhibited superior performances in promoting osseointegration at BII than the HA-coated titanium alloy at all three time periods. In particular, the sintered bionic porous titanium alloy had a high osseointegration ability, showing favorable bone ingrowth performance at 1 month. A comparison of the two porous titanium alloys showed that the sintered bionic porous titanium alloy performed better in terms of bone ingrowth and osseointegration. In this study, we observed that the porosities of the 3D group and the sintered group were 75.2% ± 1.40% and 74.3% ± 3.45%, and the pore diameters of the two groups were 532.2 ± 71.43 µm and 515.3 ± 199.49 µm. Previous studies have demonstrated that alloys with 75%–85% porosity (Mour et al., 2010) and 500 μm pore size improved osseointegration and bone formation (Fukuda et al., 2011; Wauthle et al., 2015; Chen et al., 2020). We also confirmed in our previous study that alloys with 75% porosity had a superior bone ingrowth phenomenon compared with those with 50% porosity (Li et al., 2018).

The titanium alloy forms a TiO_2_ biofilm, which can prevent infection and reduce the release of metal ions, on the surface of the body (Saini et al., 2015). Porous Ti6Al4V structures were demonstrated to be effective in supporting cell growth and new bone tissue growth, and a cell-based study suggested that Ti6Al4V possesses high cyto-biocompatibility (Chai et al., 2012; Braem et al., 2014). However, because of the increase in surface area of ​​porous Ti6Al4V, a few studies have expressed concern that these materials will increase the likelihood of metal-ion release. Thus, in our previous experiments (Li et al., 2018; Li et al., 2019a), we observed that sintered and 3D-printed materials have favorable biocompatibility and mechanical safety. The sintered porous titanium alloy may have space-holder material residues, which can cause the occlusion of pores and closure of channels, consequently affecting the effectiveness of the overall porous structure. The connectivity of pores in the porous structures provides sufficient space for fluid flow, leading to increased angiogenesis. The interconnectivity of pores is an important feature of successful osseointegration (Nguyen et al., 2012). The sintering process adopts the low-pressure slow sintering process. In this study, the electron microscope (Figure 3) and microCT (Figure 5) observations confirmed that the spatial structure was intact and unobstructed, and no distinct material residue was observed.

This study primarily compared the early bone ingrowth between the porous and HA-coated titanium alloy acetabular cup. Our previous experiments confirmed that the sintered porous titanium alloy acetabular cup exhibited a superior bone ingrowth to that of the HA titanium acetabular cup 1 year after surgery (Li et al., 2019a). However, the initial stability of the hip prosthesis is an important factor affecting the lifetime of the prosthesis; hence, in this study, we compared the bone ingrowth differences of different materials at 1, 3, and 6 months postoperatively. MicroCT scan results suggested that the sintered porous titanium alloy had a higher bone volume fraction (significant difference) than the 3D-printed titanium alloy and HA group at 1 and 3 months, and the sintered porous titanium alloy also had a higher bone volume fraction (significant difference) than that of the HA group at 6 months. The staining of the tissue sections showed an increased BIC for each material with increasing time. At 1 month after surgery, the sintered biomimetic fully porous titanium acetabular cups demonstrated strong bone ingrowth, with a large amount of bone tissue growing into the porous structure, achieving a “reinforced cement concrete-like mix” and providing favorable initial stability of the prosthesis. A favorable early bone ingrowth can help overcome the challenge of early prosthetic loosening and enable early mobilization, improving the patient’s prosthetic life and quality of life. Compared with the HA-coated titanium alloy acetabular cup, the sintered porous titanium alloy acetabular cup and 3D titanium alloy acetabular cup exhibited higher bone ingrowth. This may be due to the weak bond between the coating and the titanium interface of the HA-coated titanium cups, and the high modulus of elasticity, which leads to stress shielding. Moreover, for the currently more popular 3D-printing technology, our newly invented sintered porous titanium alloy acetabular cup exhibited a superior bone ingrowth performance. Moreover, there was no statistical difference in the bone ingrowth performance of the sintered porous titanium alloy at 1 and 6 months after surgery.

A comparison of the fluorescently labeled MAR revealed that the MAR value within the porous acetabular cup was higher than that around the cup, indicating that the porous structure was more effective in the stimulation of osseointegration within the structure than around it. In addition, studies on the distal end of the human femur have demonstrated that the MAR at the implant interface of the human trabecular bone porous coating is higher than that of the host bone in the surrounding area. Furthermore, the MAR values of the porous coating and the host bone area tend to reduce with time (Bloebaum et al., 1994). At 1 month of implantation, sintered porous titanium had a higher MAR value than those of the remaining two groups, indicating that the sintered porous structure exhibited a favorable osseointegration ability at 1 month of implantation. Consistent with the results of histology, sintered porous titanium can achieve a favorable bone ingrowth at an early stage.

In this study, 3D printing did not help achieve the osseointegration performance of the sintering process, likely because although the porosity and pore size of the two materials were not even close to statistical difference, the 3D-printed porous titanium alloy has a higher elastic modulus of 4.46 ± 0.38 GPa, whereas that of the sintered porous titanium alloy is 1.77 ± 0.28 GPa, which is closer to the elastic modulus of 1.5 GPa of trabecular bone (Brizuela et al., 2019). The closer elastic modulus of BII was associated with smaller stress shielding between the two, which is more conducive to the osseointegration of BII. Observation using a light and electron microscope (Figure 3) showed that the diameter and roughness of the wires of the two materials were different. The sintered porous titanium alloy had a thinner and rougher wire, whereas the 3D-printed wire was thicker and smoother. The *in vitro* findings of cell adhesion, proliferation, and differentiation show that the surface roughness and cell adhesion are positively correlated with the increase in osteoblastic cell activity (Velasco-Ortega et al., 2016; Pellegrini et al., 2018). Additionally, the porous structure of sintered bionic trabecular is randomly generated and considerably similar to the trabecular structure of natural trabecular bone, whereas the 3D-printed structure is prepared in a regular CAD design and does not conform to the random porous structure of natural trabecular bone. Therefore, compared with the 3D-printed porous titanium, the sintered bionic trabecular full porous titanium alloy is more identical to the natural cancellous bone structure and can achieve a strong structure like that of “reinforced cement concrete” in the early implantation stage, allowing the prosthesis to achieve early stability. Nevertheless, there are numerous 3D-printing methods, and a more optimized porous structure can be simulated on a computer to enhance bone ingrowth performance.

Thus far, numerous preclinical studies have reported on the promotion of osseointegration. Most animal studies focused on the distal femur or tibia implant (Mattila et al., 2009) for evaluating the osseointegration performance of the material, while there are some studies on the iliac bone implant of sheep (Trisi et al., 2016). Because the resting state of the implant is not stimulated by real mechanical loads in the body, the results do not fully represent the biological performance of the material in the body. Moreover, studies have demonstrated that mechanical load stimulation is an important factor affecting osseointegration, and appropriate mechanical stimulation can promote the BII osseointegration performance (Kelly and Jacobs, 2010; Ozcivici et al., 2010). THA prosthesis and matching surgical instruments suitable for beagles were used in this experiment for simulating the mechanical load borne by the hip joint prosthesis to the greatest extent, observing the differences in bone ingrowth performance of different materials, and evaluating the differences in stability of the different prostheses. BII osseointegration results under real mechanical stimulation can better demonstrate the true osseointegration performance of the material and has a more accurate guiding significance for the clinical application of sintered fully porous titanium alloy materials.

Because the same titanium alloy material (Ti6Al4V) was used for each group in this study, the difference in composition of the material–bone tissue interface was not analyzed. This study focused on optimizing the spatial structure for achieving a 3D structure that is close to that of the bone trabeculae, thus attaining a lower elastic modulus and increased osseointegration, ultimately extending the *in vivo* survival time of the artificial prosthesis. The study on the surface modification of the material, including the addition of multiple active substances for increasing the osseointegration properties of the material, is crucial. More detailed *in vitro* studies of the surface properties of the material, including roughness, are also required. These attempts are our future directions.

## 5 Conclusion

The fully porous titanium alloy prepared using the sintering process and 3D-printing technology exhibited faster and superior bone ingrowth performance than the HA-coated titanium alloy. Furthermore, the alloy prepared using the sintering process in this experiment showed a superior osseointegration performance than that prepared using the 3D-printing technology. The sintered bionic trabecular fully porous titanium alloy material provides a better option for developing joint replacement prostheses.

## Data Availability

The original contributions presented in the study are included in the article/Supplementary Material, further inquiries can be directed to the corresponding author.
