# Supplementary material for: In vivo evaluation of osseointegration ability of sintered bionic trabecular porous titanium alloy as artificial hip prosthesis
Source: Front Bioeng Biotechnol. 2022 Sep 14;10:928216. doi: 10.3389/fbioe.2022.928216 (PMC9516407; doi:10.3389/fbioe.2022.928216)
Supplement: Supplementary file 1 [file DataSheet1.PDF]

Supplementary Material

1.1 Supplementary Figures

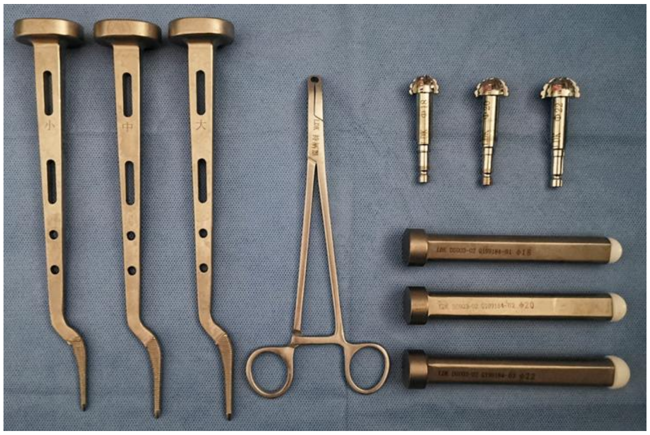

Supplementary Figure 1. Professional instruments for dogs.

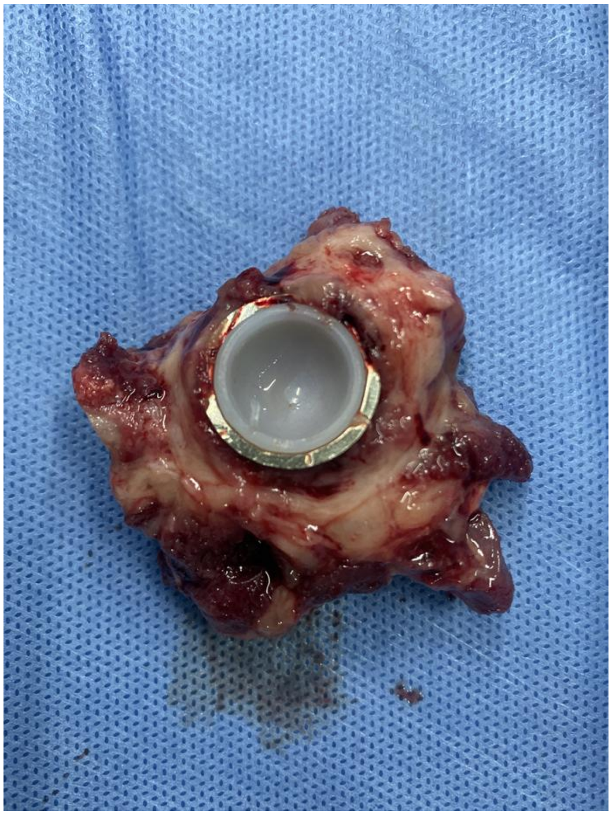

Supplementary Figure 2. Specimens of acetabular cup.

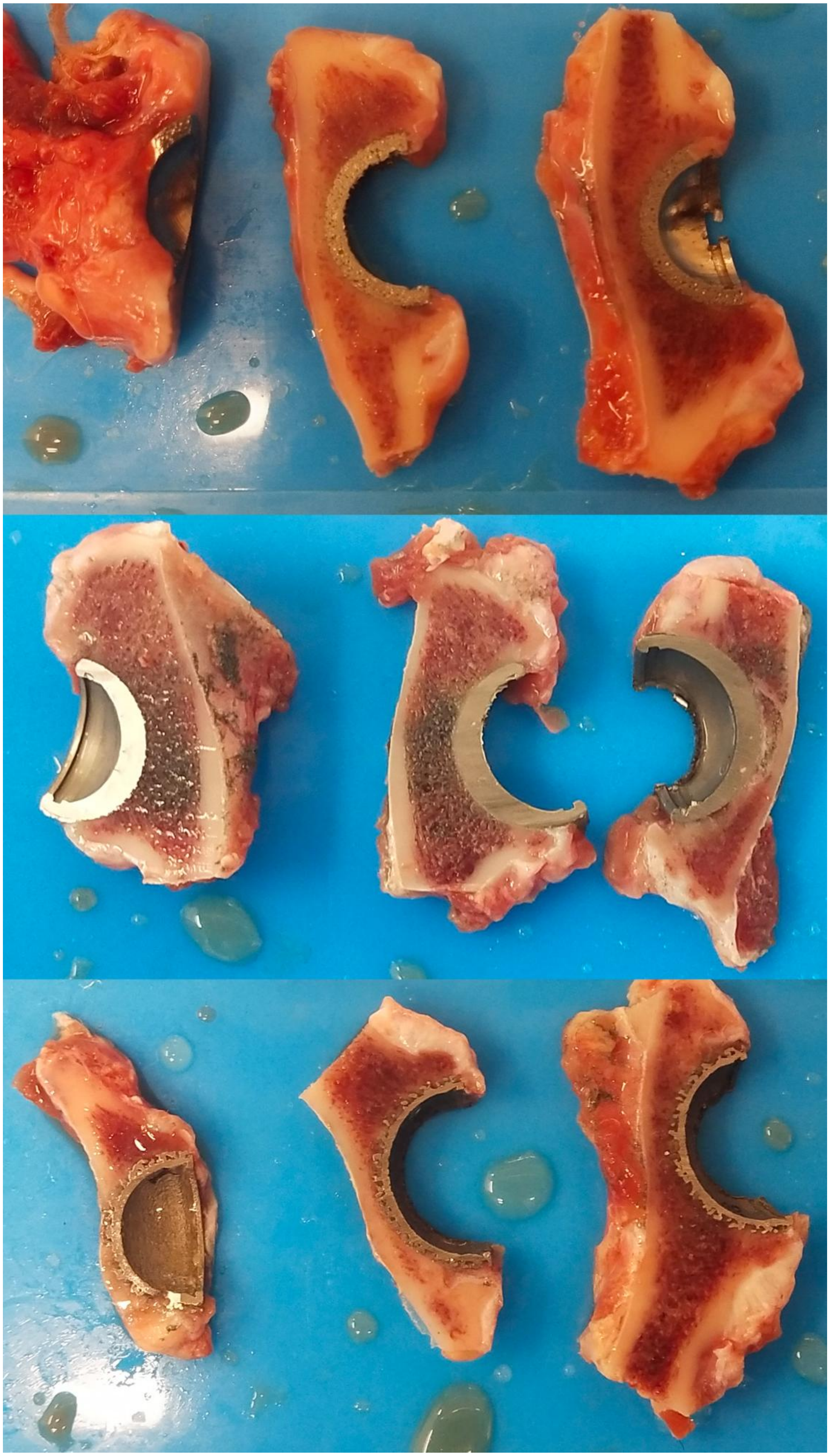

Supplementary Figure 3. The hard tissue sections.

1.2 Supplementary Videos

Supplementary video 1. The joint is reduced and examined for motion.

Supplementary video 2. Postoperative activity of animals.
